# Genomic adaptation to agricultural environments: cabbage white butterflies (*Pieris rapae*) as a case study

**DOI:** 10.1186/s12864-017-3787-2

**Published:** 2017-05-26

**Authors:** Kristin L. Sikkink, Megan E. Kobiela, Emilie C. Snell-Rood

**Affiliations:** Department of Ecology, Evolution, and Behavior, University of Minnesota, 1479 Gortner Ave, 140 Gortner Lab, Saint Paul, MN 55108 USA

**Keywords:** *de novo* transcriptome, Population divergence, Single nucleotide polymorphism

## Abstract

**Background:**

Agricultural environments have long presented an opportunity to study evolution in action, and genomic approaches are opening doors for testing hypotheses about adaptation to crops, pesticides, and fertilizers. Here, we begin to develop the cabbage white butterfly (*Pieris rapae*) as a system to test questions about adaptation to novel, agricultural environments. We focus on a population in the north central United States as a unique case study: here, canola, a host plant, has been grown during the entire flight period of the butterfly over the last three decades.

**Results:**

First, we show that the agricultural population has diverged phenotypically relative to a nonagricultural population: when reared on a host plant distantly related to canola, the agricultural population is smaller and more likely to go into diapause than the nonagricultural population. Second, drawing from deep sequencing runs from six individuals from the agricultural population, we assembled the gut transcriptome of this population*.* Then, we sequenced RNA transcripts from the midguts of 96 individuals from this canola agricultural population and the nonagricultural population in order to describe patterns of genomic divergence between the two. While population divergence is low, 235 genes show evidence of significant differentiation between populations. These genes are significantly enriched for cofactor and small molecule metabolic processes, and many genes also have transporter or catalytic activity. Analyses of population structure suggest the agricultural population contains a subset of the genetic variation in the nonagricultural population.

**Conclusions:**

Taken together, our results suggest that adaptation of cabbage whites to an agricultural environment occurred at least in part through selection on standing genetic variation. Both the phenotypic and genetic data are consistent with the idea that this pest has adapted to an abundant and predictable agricultural resource through a narrowing of niche breadth and loss of genetic variants rather than *de novo* gain of adaptive alleles. The present research develops genomic resources to pave the way for future studies using cabbage whites as a model contributing to our understanding of adaptation to agricultural environments.

**Electronic supplementary material:**

The online version of this article (doi:10.1186/s12864-017-3787-2) contains supplementary material, which is available to authorized users.

## Background

Agricultural environments have long provided an opportunity to study evolution in action [1–3], whether through adaptation to pesticides [4–6] or adaptation of pests to specific crops [7–9]. In some cases, agricultural environments may even result in diversification [10] or unique evolutionary dynamics in pests, because crop resources are incredibly abundant and homogenous relative to wild populations of resources [11–13]. Genomic tools are facilitating novel approaches to testing hypotheses about evolutionary responses of populations to agriculture [14–17]. For instance, genomic studies in aphids have shown changes in copy number, symbionts, and gene expression associated with specific crops and insecticides [18–21]. However, there have been calls to study more diverse systems in order to test a range of hypotheses about pest evolution [14].

Here, we begin to develop the cabbage white butterfly (Pieridae: *Pieris rapae*) as a new system to test questions about adaptation to agricultural environments. The cabbage white butterfly uses plants in the family Brassicaceae as hosts, which includes many cultivated species such as cabbage, canola, and radish. Thus, they are often important pests, especially for organic farmers [22–24]. Cabbage whites and their close relatives are well studied with respect to behavioral, physiological, and morphological plasticity [25–32], making them a great system to explore the relatively untested role of plasticity in the colonization of agricultural environments [14]. In the present study, we focus on the hypothesis that adaptation to a novel agricultural environment occurs at least in part through selection on standing genetic variation, resulting in a subsampling of the ancestral population. Similar to other genetic studies of adaptations of pests to agricultural environments [20, 33–35], we predict that the more recent agricultural population will show lower genetic diversity, as well as some population structure and divergence despite continued gene flow.

To test these predictions, we are studying a unique population of cabbage white butterflies that is associated with intensive canola agriculture. Northern North Dakota, southern Manitoba and Saskatchewan have been extensively farmed for canola since the late 1970s and early 1980s. In the last decade, the northeastern region of North Dakota often plants over half a million acres of canola annually [36]. Cabbage whites in this area feed on canola crops as both a larval host and adult nectar plant, especially since pesticide application is minimal and, when it does occur, is limited to early in the season when butterfly numbers are low [37]. From the perspective of pest adaptation to agriculture, this region is truly unique because in other regions of North America, Brassicaceae agriculture tends to be limited to cool seasons, whereas in North Dakota, canola is available throughout most of the flight period of cabbage white butterflies. This represents an abundant, predictable and high nutrient resource. Indeed, in some areas in the late summer, we have estimated adult butterfly density at over 150,000 individuals per hectare. Relative to other pest systems, this represents a case where an agricultural population may be adapting to a high nutrient and abundant resource without going through major pesticide-induced bottlenecks.

In contrast to this agricultural population, most populations of cabbage whites make use of many wild native and non-native mustards, in addition to using hosts in gardens, roadsides, ditches and other disturbed areas. This represents a unique opportunity to study adaptations to agricultural environments, as one can study both the agriculture-associated population and a nonagricultural population that is probably representative of the ancestral condition (found in St. Paul, Minnesota, approximately 430 miles away). Thus, at the landscape level, there is a clear mosaic of resource predictability that is likely shaping pest adaptation despite ongoing gene flow [33]. In particular, the relative homogeneity and predictability of the agricultural area should, over time, favor increased specialization and associated loss of plasticity related to the use of a range of host plants. In this research, we first describe phenotypic differences between the populations, comparing development time and adult body size of each population when reared in the lab on hosts varying in relatedness to canola. Then, we begin to develop genomic tools for comparing this unique agricultural population to nonagricultural populations. After assembling the gut transcriptome of this population, we compare patterns of differentiation in coding sequences between the two populations. We expected to find genetic differentiation between populations at both the phenotypic and genomic levels.

## Results and discussion

### Population performance on different hosts

We raised caterpillars from both the agricultural (herein referred to as ND) and nonagricultural (MN) populations under controlled conditions to contrast the performance of the two populations on hosts varying in relatedness to canola. “Canola” represents several different cultivars of three Brassica species—*Brassica napus*, *B. rapa*, and *B. juncea*. Most varieties in North Dakota are *B. napus*, but *B. rapa* is also grown [37]. We used *Brassica rapa* (var. *chinensis*) as our host approximating “canola” because canola varieties performed poorly in our greenhouse trials and were too stunted to support normal larval growth. We compared caterpillar performance on *Brassica* to that on *Raphanus sativus* (radish), both purchased as organic produce. *Raphanus* has a different profile of chemical defenses than the genus *Brassica* (glucosinolates, [38]), but is still a commonly used host in the MN population in either community gardens or as feral radish. We predicted the ND population would perform relatively better on the *Brassica* host than on *Raphanus*, as the former is more closely related to canola.

**Fig. 1:**
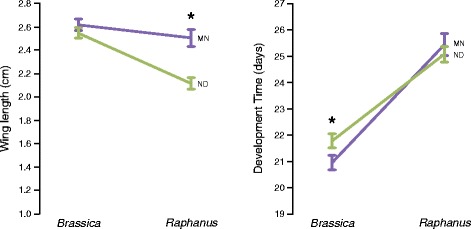
Differences in performance metrics for the nonagricultural (*purple*) and agricultural (*green*) populations when raised on different plant hosts. *Significant phenotypic differences between populations from a general linear model (*P* < 0.05)

Using general linear models that controlled for sex (Table 1), there were significant population-by-host interactions for both wing length (*P* = 0.006) and wing area (*P* = 0.009) as well as a marginally significant interaction for development time (*P* = 0.065). For both measures of body size, the ND population was significantly smaller than the MN population when raised on *Raphanus*, the host more distantly related to canola (Fig. 1). For development time, both populations developed more slowly on radish (Fig. 1), and females developed more quickly than males (Table 1, Additional file 1). Contrary to expectations, the MN population had significantly faster development time on the *Brassica* host, relative to the ND population (Fig. 1). However, the difference in development time is rather small—shifting from about 21 to 22 days—and unlikely to have a meaningful impact on fitness, especially compared to other factors. Given the extreme temperature dependence of development time, it’s also possible this trait is not the best performance measure for comparing these populations, especially given climate differences between the sites. The populations were reared simultaneously in the same climate chamber, in replicate and interspersed cups, so it is unlikely that temperature fluctuations during rearing could account for the observed difference in development time. Taken together, these results suggest that the nonagricultural (MN) population does indeed have a broader host breadth than the agricultural (ND) population, out-performing them on some metrics on host plants less closely related to canola.

**Table 1:** Larval performance on different host plant species using a general linear model

	Forewing length	Forewing area	Development time
Population	*F*_*1,58*_ = 17.4***	*F*_*1,58*_ = 17.4***	*F*_*1,58*_ = 0.49
Host	*F*_*1,58*_ = 24.2***	*F*_*1,58*_ = 27.8***	*F*_*1,58*_ = 146***
Population x Host	*F*_*1,58*_ = 8.3**	*F*_*1,58*_ = 7.2**	*F*_*1,58*_ = 3.5*
Sex	*F*_*1,58*_ = 1.6	*F*_*1,58*_ = 1.1	*F*_*1,58*_ = 6.5**

***P < 0.0001; **P < 0.01; *P < 0.10

After describing these initial differences in performance between the two populations, we performed a second, more extensive common garden experiment where we additionally harvested gut tissue for measures of gene expression (see below). In this experiment, we were prevented from doing additional phenotypic comparisons between populations because individuals from the agricultural population were more likely to go into diapause as pupae regardless of diet (80.15% from ND vs. 0% from MN, N = 258, Χ^2^ = 215.9, *P* < 0.0001). This suggests additional genetic differentiation between the populations – the agricultural population from northern North Dakota may have a different threshold of diapause induction due to the shorter growing season relative to southern Minnesota or the harvesting of canola in mid-August. Comparing agricultural and nonagricultural populations at the same latitude would help to distinguish these two hypotheses. While this population difference in diapause induction may represent an adaptation to abiotic conditions rather than an adaptation to an agricultural environment per se, it still represents a significant difference between the two populations in a common garden.

Our controlled laboratory experiments suggest significant phenotypic differentiation in fitness proxies between these agricultural (ND) and nonagricultural (MN) populations of cabbage whites on the two hosts. Most striking is the reduced body size observed in the ND population when raised on the nonagricultural host plant, which suggests that the ND population has a reduced capability to utilize suitable host plants outside of the genus *Brassica*. Such changes are likely associated with genetic differentiation in genes related to larval feeding. Future experiments comparing the niche breadth of these two populations would be strengthened by including North Dakota canola varieties and a range of other host plants. Given our struggles growing high quality canola in greenhouse conditions, this would likely be best accomplished with organic agricultural plots of a range of host plants, with leaves harvested daily for lab rearing in common climatic conditions.

### Transcriptome assembly

There are currently limited genomic resources for *Pieris rapae* to facilitate studies that investigate the genetic basis of the adaptation to specific host plants, such as we observe in the agricultural population. Although transcriptome assemblies have recently been completed for *Pieris rapae* [39, 40], these studies had limited representation of the caterpillar stages and tissues that are relevant to address the hypothesis that changes in gene expression in the gut contribute to adaptation to agricultural environments. To address this limitation, we set out to more fully characterize the transcriptome in the digestive tract of *Pieris rapae* caterpillars.

To do this, we collected gut tissue from 6 descendants of butterflies collected from the ND population reared on either *Brassica* or *Raphanus*. We chose to focus on a single population in order to minimize the genetic variability, thus simplifying the transcriptome assembly. Of these, four caterpillars were collected at the 5^th^ instar stage, and two were collected as 2^nd^ instars. In addition, for one of the 5^th^ instar larvae, we also sequenced RNA from the fat body, a tissue that also plays a critical role in insect metabolism and energy storage [41]. All samples were sequenced in a single lane of an Illumina HiSeq2000. A summary of the combined sequencing results is presented in Table 2.

**Table 2:** Summary of sequencing and transcriptome assembly results

Sequencing (for *de novo* transcriptome)	
Raw reads (101 nt paired-end)	179472918 pairs
Cleaned reads	159812653 pairs
	14974970 orphans
Sequenced bases (cleaned)	31.8 Gb
Assembly	
Number of transcripts (contigs)	31624
Number of unigenes	17595
Mean length (unigene)	1420.69 bp
Median length (unigene)	909 bp
N50 (unigene)	2416 bp
Assembled length (unigenes)	25.0 Mb
GC content (unigenes)	38.8%
Number of protein-coding ORFs	13991
Mean ORF length	1058.4 bp
BUSCO arthropod gene set (2675 genes)	
Complete, single-copy	1875 (70.1%)
Complete, duplicated	72 (2.7%)
Partial	165 (6.2%)
Missing	563 (21.0%)

We assembled high-quality sequencing reads using the Trinity transcriptome assembly platform [42, 43]. After filtering small and fragmented contigs, and removing genes that matched plant or bacterial contaminants, the resulting assembly contained 31,624 contigs (*i.e.* transcripts) from 17,595 unique clusters (unigenes). From each transcript cluster, we selected the contig with the longest predicted open reading frame (ORF) to represent the consensus sequence of the unigene. Unless otherwise noted, all analyses were performed on the set of consensus unigene sequences, to minimize the probability that a given gene was represented multiple times in each statistic. The characteristics of the final transcriptome assembly are summarized in Table 2.

The final unigene sequences were compared against the arthropod Benchmarking Universal Single-Copy Orthologues (BUSCO) [44]. The arthropod BUSCOs are a set of 2675 proteins that are expected to be present as a single-copy gene in all arthropod species, and can be used as a benchmark for assessing the completeness of a gene set. A significant fraction (21.0%) of BUSCO genes were not found among the *Pieris rapae* unigenes, likely because our transcriptome was assembled from a narrow range of tissues and stages. Of the BUSCOs that were matched to assembled unigenes, the majority were found in a single copy, and most recovered the complete protein sequence (Table 2). Thus, despite using a limited set of tissues and developmental stages, we have nevertheless assembled a high quality transcriptome that covers a significant fraction of the expected genes in *Pieris rapae*.

### Transcriptome annotation

Most of the assembled transcripts show significant sequence similarity to existing protein databases (Table 3), indicative of the high quality of our final assembled transcripts. Of the representative sequences selected for each unigene, 11,049 (62.8%) showed significant sequence similarity (BLASTx, E value < 10^-5^) to the silk moth (*Bombyx mori*) protein database. Similarly, a majority of unigenes (70.2%) showed significant similarity to proteins in the NCBI non-redundant (nr) protein database (BLASTx, E value < 10^-5^). Fewer (51.3%) matched *Drosophila* proteins, likely due to the longer divergence time from Lepidoptera. Nearly half of the assembled sequences matched entries in the high-quality, manually curated Swiss-Prot protein database [45].

**Table 3:** Summary of *Pieris rapae* transcriptome annotation

Unigene annotation	
*Bombyx mori* proteins (E < 10^-5^)	11,049 (62.8%)
*Drosophila melanogaster* proteins (E < 10^-5^)	9041 (51.3%)
NCBI nr database (E < 10^-5^)	12,358 (70.2%)
UniProt/Swiss-Prot database (E < 10^-5^)	8651 (49.2%)
UniProt/TrEMBL database (E < 10^-5^)	12,230 (69.5%)
InterProScan	11,537 (65.6%)
Pfam domain^a^	11,428 (65.0%)
GO annotation^b^	7464 (42.4%)
KEGG annotation^a^	4554 (25.9%)

^**a**^Pfam and KEGG searches included only sequences from 13979 protein coding ORFs

^**b**^GO matches were identified for 9595 unigenes, of which 7464 met significance cutoff requirements for annotation

Using the significant hits to the nr protein database, we used the functional mapping software Blast2GO [46–48] to assign gene ontology (GO) terms from the generic GO-Slim dictionary [49, 50] to the unigenes in our *Pieris rapae* transcriptome. In all, 7464 unigenes (42.4%) were successfully annotated with at least 1 GO term using default parameters for annotating GO matches. As expected, we saw many genes mapping to biological process terms such as carbohydrate metabolic process (215 unigenes), lipid metabolic process (194), or more generally to biosynthetic process (832 unigenes) (Additional file 2). Other key functions of the gut, including transport (269), response to stress (225), immune system function (59), and homeostatic processes (80), were also represented in the expressed genes. The molecular function term with the highest representation in our transcriptome was ion binding (2092), followed by oxidoreductase activity (506).

**Fig. 2:**
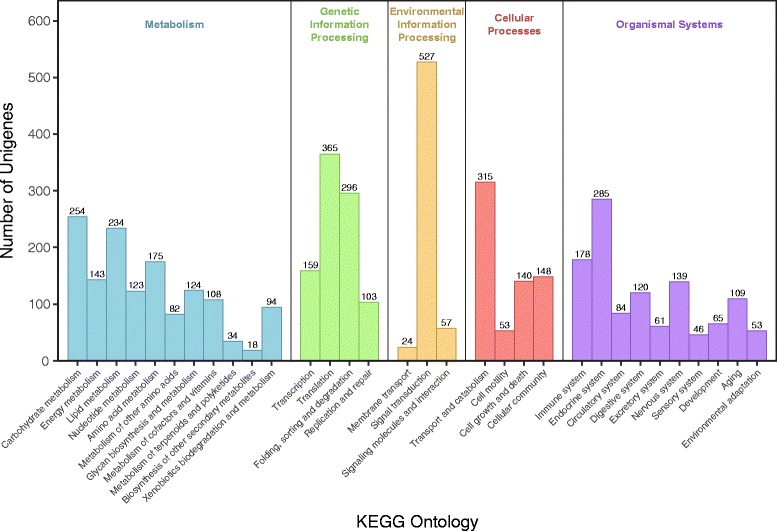
Number of annotated unigenes assigned to major KEGG ontologies from the *Pieris rapae* transcriptome

We also used BlastKOALA [51] to assign our genes to pathways in the KEGG ontology [52, 53]. 4554 unigenes (25.9%) could be assigned to KEGG pathways (Fig. 2; Table 3). Many genes were annotated as belonging to at least one of the major metabolic pathways, including carbohydrate, lipid, and amino acid metabolism. The largest KEGG category was the signal transduction proteins, of which we annotated 527 genes. Notably, we identified components of several organismal system pathways, including digestive and excretory systems, immune system, and development, which we expect to be expressed in gut tissue or at early developmental stages.

### Population divergence in gut transcripts

To examine population differences between ND and MN populations of *Pieris rapae*, we collected gut RNA from early and late stage larvae from each of the two populations, and scanned for polymorphic loci that were divergent between the two populations. We identified 63,595 highly supported biallelic single nucleotide polymorphisms (SNPs) present in at least 16 individuals from each population in the *Pieris* gut transcriptome, after applying stringent quality filters to minimize the impact of sequencing errors and misalignments. These SNPs were located on 5105 unigenes, with a polymorphism rate of 5.5 SNPs per kb of sequence within the variable genes (Table 4).

**Table 4:** Summary of biallelic SNPs identified in the final transcriptome assembly

SNP summary	Total	Filtered^a^
Number of biallelic SNPs	524184	63595
SNPs/bp (all unigenes)		0.0025
Number of unigenes containing variants		5105
SNPs/bp in variant unigenes		0.0055
% transitions^b^		60.55%
% in predicted coding transcripts		98.59%
% in exons		71.51%
% nonsynonymous substitutions		12.50%
% synonymous substitutions		59.01%

^**a**^SNPs passing filtering criteria have a called genotype (Q > 20) in at least 16 individuals per population with a minor allele frequency >1%

^**b**^Expect 33% if transitions occur at random

The two populations showed very little genetic differentiation across the transcriptome. The average differentiation between the two populations, measured by G_ST_ [54], was 0.018 across all loci. Furthermore, we did not find any sites with fixed differences between the ND and MN populations (Additional file 3). Our observation of very little differentiation (F_ST_ < 0.05; [55]) is comparable to other population genetics studies in butterflies, most of which find little genetic differentiation [56–60] or only modest differentiation (F_ST_ < 0.10; [59, 61–63]) in either microsatellites or allozymes at a variety of spatial scales. As a species, *Pieris rapae* is highly mobile, and will readily disperse over significant distances [64], and may also make use of a variety of fragmented habitats, like the closely related *Pieris napi* [59]. These traits may contribute to the low population differentiation overall by enabling gene flow across Minnesota and North Dakota.

**Fig. 3:**
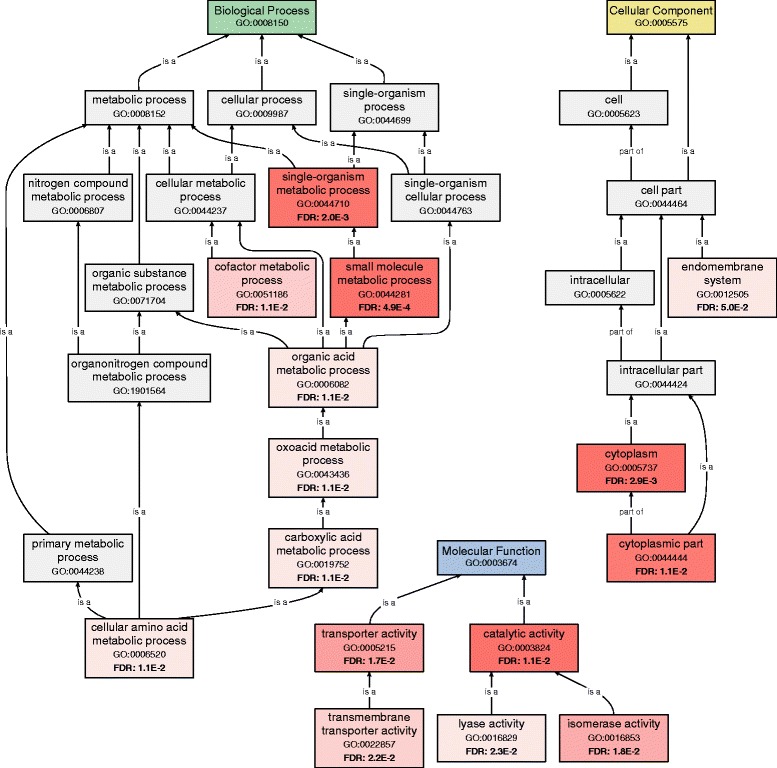
GO categories enriched among genes containing significantly differentiated SNPs between populations. Red shading signifies significant enrichment of genes mapping to the GO term (FDR<0.05). The intensity of the shading scales with the significance of that term

Despite the low overall divergence between the agricultural and nonagricultural populations, there were 318 SNPs on 235 unique genes that were significantly differentiated between populations (Fisher’s exact test, Bonferroni corrected p < 0.05). Values of G_ST_ for the significantly differentiated SNPs ranged from 0.11 to 0.29 (see Additional file 4). The set of unigenes containing significant SNPs was significantly enriched for cofactor and small molecule metabolic processes (FDR < 0.05). Many of the genes in this set also have transporter or catalytic activity from the molecular function ontology tree (Fig. 3; Additional file 4).

Most of the significantly differentiated SNPs were synonymous coding changes (200). Only two unigenes lacking predicted ORFs differed between the two populations. 83 SNPs fell in UTR regions flanking a predicted ORF. Some of these may include regulatory changes that alter expression levels of the associated transcripts. Finally, 33 nonsynonymous changes were significantly different between populations.

The set of 29 genes containing divergent nonsynonymous SNPs are of particular interest as candidate genes that could facilitate adaptation to different host plant environments (Table 5). Three unigenes of particular interest are c15052_g1, c16008_g1, and c18004_g1, all of which contain multiple nonsynonymous SNPs. Unigene c16008_g1 contains significant sequence similarity to nuclear pore complex protein Nup50, and has annotated functions in protein transport and neurological system processes. Unigene c18004_g1 is most similar to phosphatase I regulatory subunit 15a, which in mammals may facilitate recovery after cellular stress [65]. Other genes with significant nonsynonymous SNPs are involved in a variety of metabolic processes (Table 5).

**Table 5:** Summary of 33 nonsynonymous SNPs that are significantly different between populations

Unigene	Position	G_ST_	G’_ST_	D	ϕ_ST_	Description	GO annotation^a^
c5933_g1	548	0.14	0.33	0.12	0.58	Transport and Golgi organization 11	C:cellular_component; F:isomerase activity
c7209_g1	1211	0.15	0.44	0.23	0.53	FK506-binding 59 isoform X1	P:protein folding
c10952_g1	794	0.18	0.44	0.19	0.51	Ankyrin repeat domain-containing 11	C:cellular_component
c11274_g1	630	0.17	0.43	0.19	0.54	UDP-galactose 4-epimerase	P:biological_process; F:molecular_function
c11590_g2	235	0.14	0.33	0.10	0.38	BCL-6 corepressor 1	
c11789_g1	423	0.14	0.38	0.17	0.45	hypothetical protein KGM_20694	C:cellular_component
c11993_g1	616	0.16	0.39	0.15	0.60	alpha-tocopherol transfer -like	P:transport; F:transporter activity; C:intracellular
c12584_g1	1268	0.13	0.37	0.18	0.44	PREDICTED: uncharacterized protein LOC106138810, partial	
c12711_g1	1584	0.16	0.35	0.11	0.53	Recombination repair 1	C:nucleus; F:DNA binding; F:nuclease activity; F:lyase activity; F:ion binding; P:DNA metabolic process; P:response to stress
c13334_g1	1471	0.16	0.45	0.24	0.49	aspartate--tRNA ligase, cytoplasmic	C:cytoplasm; F:ion binding; P:tRNA metabolic process; P:cellular amino acid metabolic process; F:ligase activity; P:translation
c13731_g1	1607	0.13	0.35	0.14	0.51	Chorion b-ZIP transcription factor	F:DNA binding; F:nucleic acid binding transcription factor activity; P:cellular nitrogen compound metabolic process; P:biosynthetic process
c14347_g1	644	0.11	0.26	0.06	0.46	aldehyde dehydrogenase X, mitochondrial-like	F:oxidoreductase activity
c15052_g1	791	0.16	0.35	0.12	0.43	glutamic acid-rich -like	P:biological_process; C:extracellular region; F:molecular_function
c15052_g1	873	0.15	0.37	0.14	0.45	glutamic acid-rich -like	P:biological_process; C:extracellular region; F:molecular_function
c15359_g2	775	0.13	0.28	0.08	0.38	PREDICTED: uncharacterized protein LOC101740601	C:membrane; C:integral component of membrane
c15377_g1	1122	0.19	0.43	0.16	0.53	prion-like-(Q N-rich) domain-bearing 25	C:membrane; C:integral component of membrane
c15434_g1	67	0.15	0.44	0.25	0.56	cholinesterase 1-like	P:metabolic process; F:hydrolase activity
c15846_g1	1999	0.16	0.39	0.16	0.63	nicastrin	C:cellular_component
c15990_g1	1062	0.14	0.42	0.24	0.51	pancreatic triacylglycerol lipase-like	P:biological_process; C:extracellular region; F:molecular_function
c16008_g1	531	0.22	0.55	0.30	0.71	nuclear pore complex Nup50	C:nuclear envelope; P:nucleocytoplasmic transport; P:protein targeting; P:vesicle-mediated transport; P:signal transduction; P:cell differentiation; P:anatomical structure development; P:neurological system process; F:molecular_function
c16008_g1	789	0.24	0.58	0.32	0.78	nuclear pore complex Nup50	C:nuclear envelope; P:nucleocytoplasmic transport; P:protein targeting; P:vesicle-mediated transport; P:signal transduction; P:cell differentiation; P:anatomical structure development; P:neurological system process; F:molecular_function
c16008_g1	853	0.22	0.49	0.21	0.73	nuclear pore complex Nup50	C:nuclear envelope; P:nucleocytoplasmic transport; P:protein targeting; P:vesicle-mediated transport; P:signal transduction; P:cell differentiation; P:anatomical structure development; P:neurological system process; F:molecular_function
c16231_g1	2477	0.19	0.45	0.19	0.74	serine palmitoyltransferase 1	F:ion binding; P:biosynthetic process; C:cellular_component
c16497_g2	1985	0.18	0.44	0.20	0.57	adenosylhomocysteinase	P:sulfur compound metabolic process; P:cofactor metabolic process; C:cytosol; P:cellular amino acid metabolic process; P:cellular nitrogen compound metabolic process; F:molecular_function
c16508_g2	391	0.12	0.32	0.12	0.57	phosphoenolpyruvate carboxykinase	P:small molecule metabolic process; P:carbohydrate metabolic process; F:lyase activity; F:ion binding; P:biosynthetic process; F:kinase activity
c16613_g1	749	0.18	0.41	0.14	0.65	translocator -like isoform X2	C:intracellular
c17019_g1	1437	0.16	0.44	0.23	0.54	FAM114A2 isoform X1	
c17117_g1	1294	0.13	0.32	0.11	0.57	saccharopine dehydrogenase-like oxidoreductase	F:oxidoreductase activity; C:cellular_component
c17365_g1	1932	0.14	0.34	0.12	0.49	otopetrin-2-like isoform X1	C:cellular_component
c17954_g2	1970	0.15	0.36	0.12	0.61	probable uridine nucleosidase 2 isoform X2	P:metabolic process; F:hydrolase activity
c18004_g1	465	0.22	0.56	0.32	0.67	phosphatase 1 regulatory subunit 15A	C:cellular_component
c18004_g1	566	0.18	0.48	0.26	0.59	phosphatase 1 regulatory subunit 15A	C:cellular_component
c18452_g1	386	0.14	0.31	0.08	0.55	serine-rich adhesin for platelets-like isoform X1	F:calcium ion binding; C:membrane; C:integral component of membrane; P:cell adhesion; P:homophilic cell adhesion via plasma membrane adhesion molecules; C:plasma membrane

^a^GO descriptions are designated as cellular component (C), molecular function (F), or biological process (P)

A few other genes stand out as being potentially related to the difference in the onset of diapause in the two populations or tolerance to a colder winter in North Dakota. Unigene c13176_g2, identified as heat shock 70 cognate 3, has 8 significantly differentiated synonymous SNPs, as well as an additional sequence change in the 3’ untranslated region (UTR) of the gene. This cognate has been shown to respond to cold shock in other insects [66]. Furthermore, two unigenes with nonsynonymous mutations (c16497_g2 adenosylhomocysteinase and c14347_g1 aldehyde dehydrogenase X, mitochondrial-like) are, among other functions, part of the juvenile hormone pathway in insects [67, 68], and thus we speculate could be involved with the differences in diapause onset or development time between these two populations [69].

Finally, we also estimated the allele frequency spectrum for the variable unigenes using Tajima’s D [70] (Additional file 5). Of the genes with significantly differentiated SNPs, 20 genes (8.5%) had values of Tajima’s D that differed substantially from the expectation of neutrality in at least one of the two populations. In most cases, Tajima’s D was negative, suggesting that positive selection may be acting on these genes. While we cannot rule out the possibility that demographic processes may account for these observations [71], these genes are nevertheless excellent candidates for future studies.

### Complex population structure in *Pieris*

**Fig. 4:**
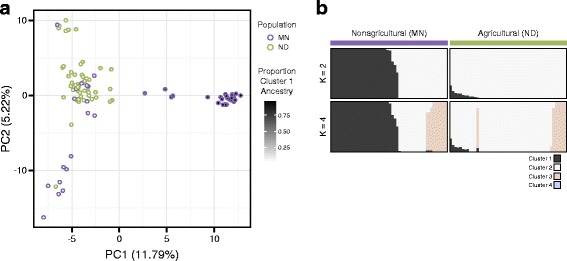
**a** Principal components analysis using the subset of SNPs genotyped in all individuals. Points are shaded according the proportion of ancestry from Cluster 1 for each individual in the fastStructure analysis with *k* = 2. **b** FastStructure analysis based on all SNPs for *k* = 2 and *k* = 4

We next performed principal components analysis to examine patterns of population structure in more detail for our two sampled populations (Fig. 4a). For this analysis, we included only SNPs that could be genotyped for all 96 individuals (2287 SNPs in total). Surprisingly, the two populations were not clearly distinct from one another on the major axes of variation, although this may be consistent with the observation of rather low differentiation between the populations. In MN, about 25% of the individuals clustered with the ND population on PC1 and PC2. However, the rest of the MN caterpillars clustered separately on either PC1 or PC2, but not both.

To investigate this pattern further, we used fastStructure [72] to examine population structure on the full set of SNPs. At a value of *k* = 2, fastStructure identified 2 apparently distinct subpopulations within the nonagricultural MN population (Fig. 4b). The first subpopulation clusters with the agricultural ND population. The second subpopulation occurs almost exclusively within the MN population, although there is a small amount of introgression in a few of the ND individuals. In fact, the individuals in this MN-specific subpopulation also have high scores on PC1, confirming our observations of population structure within MN. Additional substructure on PC2 can be detected using *k* = 4. These analyses of population structure suggest that the ND population contains only a subset of the genetic variation present in the MN population. This is consistent with the hypothesis that at least some adaptation to the agricultural environment in North Dakota resulted from selection on standing genetic variation.

We also considered the alternate hypothesis that some of the structure in the nonagricultural population could be a consequence of having sampled siblings, which was a possibility with our egg collection setup that harvested eggs from large groups of females. We examined genetic relatedness among individuals within each of the populations to determine whether the observed population structure could be explained by the presence of a single full-sibling family with an unusual genetic background. In both populations, we identified several clusters of two or more individuals that are likely to be full siblings, and many of the putative sibling pairs were also laid on the same or consecutive dates, further supporting the likelihood of the full-sibling relationship (Additional file 6). In MN, we also identified a large cluster of individuals that corresponded to the second subpopulation identified in the fastStructure analysis. While these individuals tend to be more genetically related than the rest of the nonagricultural population, most did not seem to be full-siblings. Furthermore, these individuals were laid during two distinct collection periods, with non-overlapping sets of mothers. It is therefore unlikely that the pattern can fully explained by the influence of a single family. In other words, it is unlikely that a single female dominated egg laying in a group cage and biased the results. Behavioral observations of egg-laying cages are also consistent with this interpretation – multiple females are often observed laying simultaneously in group cages.

## Conclusions

This research represents the first step in developing cabbage white butterflies as a case study to contribute to our understanding of adaptation to agricultural environments. The present results are consistent with the idea that adaptation to a novel agricultural environment occurred via selection on existing genetic variation in the ancestral population. The performance differences of the two populations on different hosts suggests that the agricultural population has become more specialized; the genetic data suggests this specialization may have occurred through selection on a subset of standing variation rather than *de novo* gain of adaptive alleles. In future work, we plan to more explicitly test the role of plasticity in this process. Plasticity is thought to enable the colonization of novel environments, including cities and agricultural monocultures [73–76]. In predictable environments such as monocultures, any costs associated with plasticity could result in the loss of plasticity and the fixation of traits following colonization [77–79]. Genomic approaches can facilitate tests of such “genetic assimilation” by testing the prediction that developmental pathways expressed by the ancestor become constitutively expressed in derived populations [80–82].

## Methods

### Population adaptation to hosts

In 2011, we first performed a common garden rearing experiment to test for differences between the agricultural (ND) and nonagricultural (MN) populations in host performance. Approximately 40 females were collected adjacent to canola fields in Cavalier and Ramsey County, North Dakota, USA, which has been a center of canola agriculture for 30 years (see additional details in introduction). Approximately 30 females were collected from roadsides, community gardens, and campus agricultural fields in St Paul, Minnesota, USA.

Larvae were reared in 16 oz. plastic containers in groups of 2-6 individuals (average 5.5). Larvae were provided with either *Brassica rapa* (var. *chinensis*), a host closely related to canola, or *Raphanus sativus* (radish), both purchased as organic produce and refreshed daily. We initially sought to rear individuals on canola varieties grown in North Dakota, but these varieties performed poorly in greenhouse conditions. Larvae were reared in a climate chamber at 24°C with a 14 hour day length. Populations were reared simultaneously in the same climate chamber, in replicate and interspersed cups. Wing length, wing area, and development time (egg to adult emergence) were taken as measures of performance. To measure wing traits, we removed forewings from individuals with forceps, photographed wings and measured length and area using the line and polygon functions in Image J (NIH). Wing length was defined as the distance from the articulation point of the forewing with the thorax to the wing apex. Measures of body size and development time were analyzed for 25 MN individuals and 38 ND individuals randomly selected from a wide range of rearing cups.

Phenotypic traits were compared using general linear models in JMP 13 (SAS Institute) using population, host, population x host, and sex as independent variables. Because males have larger wings than females, we controlled for sex in the analysis. We only included one interaction as we had *a priori* reason to suspect interactions between population and host. Post hoc analysis showed no significant interactions between population and sex.

### Population sampling for transcriptome and SNP calling

In mid- to late-August 2014, we resampled the same field sites used in the 2011 rearing experiment. Approximately 60 females were collected from ditches and roadsides adjacent to canola fields in Cavalier and Ramsey County, North Dakota, USA. For the nonagricultural population, approximately 30 females were collected from roadsides, community gardens, and campus agricultural fields in St Paul, Minnesota, USA.

Females were set up in groups of 4-10 for egg harvesting in replicate population cages (61x61x61 cm “bug dorm” tent cages) in a greenhouse. Each cage contained one *Brassica rapa* (var *silvestris*) and one *Raphanus sativa* (var *Hong Vit*), changed out daily, for egg collection. Females had access *ad libitum* to a 10% honey solution on 3-4 yellow sponges set in small petri dishes. Humidity in cages was kept elevated by the plants and wet towels on which they were set. Supplemental lighting was provided in the greenhouse to extend the day length to 15 h. Eggs were collected between August 22 and September 1. In total, eggs were obtained from approximately 27 ND females and approximately 17 MN females (based on the number of females rotated into cages that were alive for at least 24 h of egg collection). Spermatophore counts (N = 16 females) revealed that females mated on average with 1.6 males in the MN population and 1 male in the ND population.

### Tissue harvesting for RNA sequencing

Larvae for the transcriptional profiling experiments were reared on either *Brassica rapa* (var *silvestris*) or *Raphanus sativa* (var *Hong Vit*), spread across 33 replicate mesh cages. In addition, a subset of larvae from each population was reared on one host type before being switched to the alternate host at the 3^rd^ instar stage. This combination of diet regimes was selected to capture a range of transcriptional responses relevant to larval development and gut function. We harvested larval tissue at two stages for RNA sequencing (RNA-seq): 2^nd^ instar larvae harvested 7 days after egg laying, and 5^th^ instar larvae harvested 17 days after egg laying. Parallel comparisons of phenotypic adaptation to host plant were not possible in this experiment given that a large proportion of ND larvae went into diapause under greenhouse rearing conditions and diapaused individuals were significantly smaller than non-diapaused individuals.

For each larva, we harvested midgut tissue in RNase-free conditions. For the 5^th^ instar larvae, we also harvested fat body tissue, which is important for metabolism and energy storage [41]. We avoided any 5^th^ instar larvae in the wandering stage (soon to pupate) and aimed to dissect an equal number of males and females based on the presence of paired, dorsal testes. Excised tissue was placed in 350 ul of RLT Plus buffer (Qiagen, Hilden, Germany) containing 2-mercaptoethanol. Samples were placed immediately on ice prior to maceration with a pestle and flash freezing. All samples were stored at -70°C until RNA isolation. A small subset of these samples from the ND population was sequenced for the transcriptome assembly as described below. A larger set of 96 samples including individuals from both populations was used for SNP discovery.

### RNA extraction and sequencing

#### Transcriptome assembly

To construct a high-quality transcriptome assembly, we sampled individuals from the ND agricultural population only. We focused on the ND population for the reference assembly because we predicted that this population would have less genetic variation and would therefore result in a higher quality transcriptome. We included 2 gut samples and 1 fat body sample from 5^th^ instar larvae fed on *Brassica rapa*, 2 gut samples from 5^th^ instar larvae fed on *Raphanus*, and 2 gut samples from 2^nd^ instar larvae fed on *Brassica*. Total RNA was isolated from dissected gut or fat body tissue using an RNeasy Plus Mini Kit (Qiagen) according to the manufacturer’s directions. Libraries enriched for mRNA were prepared using TruSeq RNA Library Preparation Kit v2 (Illumina, San Diego, CA, USA) at the University of Minnesota Genomics Center (UMGC; Minneapolis, MN). All samples were sequenced in a single lane on an Illumina HiSeq 2000 at UMGC. In total, we generated 179.5 million paired-end reads 100 bp in length (Table 1) that were used for the transcriptome assembly.

#### SNP discovery

To examine population differences between MN and ND populations of *Pieris rapae*, we collected gut samples from 2^nd^ instar and 5^th^ instar larvae reared on *Raphanus*, *Brassica*, or both as described previously. In total, we collected tissue from 48 individuals from each population for SNP discovery (N = 8 per treatment). All mRNA libraries were prepared using the TruSeq kit as described above. The samples used for SNP discovery were sequenced across 4 lanes on an Illumina HiSeq 2500 (high-output mode) at UMGC. A total of 1118.3 million single-end reads, 50 bp in length, were generated.

### Transcriptome assembly and annotation

After demultiplexing, all sequenced reads were cleaned and trimmed using Trimmomatic (version 0.33) [83]. Sequences containing TruSeq adapter sequences were trimmed, as were low quality (Q < 5) bases from the beginning or end of the reads. We also applied a sliding window filter, so that reads were trimmed after the average quality for each 4-bp window reached Q < 20. Any reads that were shorter than 36 bp in length after trimming were discarded.

Cleaned and trimmed reads were assembled using the Trinity *de novo* assembler (version r20140717) [42, 43]. During assembly, we utilized the *in silico* normalization option in Trinity to improve memory usage. In addition, we required a minimum k-mer coverage of 2, which improved the quality of the preliminary assembly by reducing sequencing errors.

We further refined the initial assembly with several stages of filtering. First, we removed redundant contigs (contigs which were completely overlapping, with 100% sequence identity) with CD-HIT-EST (version 4.6.1) [84]. Next, we aligned the original reads from the ND samples to the assembled transcriptome using bowtie2 (version 2.2.4) [85]. Of the reads from the ND population that were used, 86.78% aligned back to assembly. Any contigs that had no reads aligned to them were removed from the final assembly. Finally, we used TransDecoder (version 2.1.0) [86] to find the longest predicted open reading frame for each contig. Contigs with fragmented ORFs less than 150 nucleotides (50 amino acid residues) in length were removed from the final assembly. We also used TransDecoder to identify genes predicted to code for functional proteins (see below). However, if transcripts were not predicted to contain a protein-coding ORF, they were retained in our assembly as “non-coding” transcripts, as long as they also met the minimum transcript length requirement.

Finally, we used a translated BLAST query (blastx, BLAST+ version 2.2.29) [87–89] to compare the unigenes to the NCBI nr database. We identified 5 unigenes with significant sequence similarity (E < 1e-5) to the host plant genera *Brassica* and *Raphanus*, as well as the related *Arabidopsis*, also in the plant family Brassicaceae, but no similarity to any species within Insecta. These sequences are likely to represent a low level of contamination from the caterpillars’ food source, rather than caterpillar-expressed transcripts. Therefore, all transcripts resulting from these unigenes were removed from the final version of the *Pieris rapae* transcriptome. Additional contamination screening identified 14 unigenes including bacterial and Illumina primer contaminants that were also removed or trimmed in the final assembly.

We selected a single representative transcript for each unigene to use as the consensus sequence for further annotation and SNP analysis, so that each unigene would only appear once. For genes that were predicted to have a protein-coding ORF by TransDecoder, we selected the transcript for each unigene that had the longest ORF, and longest transcript length, to serve as the consensus sequence. If the gene did not have a predicted likely coding region, we selected the longest assembled transcript for the cluster.

We used the BUSCO software pipeline (version 1.1b1) [44] to assess the completeness of our final transcriptome. The consensus sequences of the unigenes were mapped against the arthropod BUSCO gene set.

### Transcriptome annotation

We used a translated BLAST query (blastx) [87–89] to identify homology to genes in the *Bombyx mori* [90, 91] and *Drosophila melanogaster* [92] protein databases, the UniProtKB/Swiss-Prot and UniProt/TrEMBL protein databases [45], and the NCBI nr protein database. Transcripts were determined to have significant homology if E < 1E-5.

Using the significant blastx hits to the nr protein database, we mapped gene ontology (GO) terms [49, 50] to transcripts with the software package Blast2GO (version 3.3.5) [46–48] using default parameters for annotating GO matches. The GO terms used were a subset of terms taken from the generic GOSlim ontology dictionary. Blast2GO was also used to identify protein families and domains from the InterPro database [93].

Finally, we used BlastKOALA [51] to assign unigenes to pathways in the Kyoto Encyclopedia of Genes and Genomes (KEGG) ontology [52, 53]. Query sequences were searched against the genus_eukaryotes KEGG GENES database for K number assignment.

### Variant discovery and annotation

Demultiplexed sequence reads from the 96 samples used to examine population divergence were cleaned and trimmed as described above. Reads were then aligned to the *Pieris rapae* gut transcriptome assembly with bowtie2 [85] with the “sensitive” preset parameter options. To simplify the downstream analysis, only the representative consensus sequence for each unigene was used as reference for the alignment.

We performed SNP and indel discovery and genotyping across all 96 samples simultaneously with the Genome Analysis Toolkit (GATK; [94]) using hard filtering parameters appropriate for RNA-seq data. Prior to variant discovery, reads in regions identified as possible indels were realigned according to GATK Best Practices recommendations [95, 96]. Because the distribution of RNA-seq reads does not match the expectation of genome wide sequence reads, we chose not to filter reads tagged as duplicates. However, the SNP sets obtained with and without the duplicate read filter were very similar—87.4% of the SNPs we identified in our data were also present when the duplicate reads were removed.

Genetic variants were identified using GATK HaplotypeCaller. The minimum phred-scaled confidence threshold for calling and emitting variants was set to 20. We chose to focus on biallelic SNPs, since calling indels may be complicated by differential splicing patterns in genes, especially given that we are only focusing on a single representative transcript for each potential unigene. We filtered called biallelic SNPs using the following criteria based on current recommendations from GATK’s Best Practices recommendations for RNA-seq data: SNPs were removed if they had a FisherStrand score greater than 30, if the depth-corrected quality score of the variant call was less than 2, and if there were more than 3 SNPs within a 35bp window. In addition, we required a sequence depth of at least 25 reads at each SNP. Among the SNPs passing these filters, we required that individual genotype calls have a quality score of at least 20—individuals with a low quality genotype at a given SNP were marked as “no call” for that SNP. After removing low quality individual genotype calls, we removed SNPs that were present in less than 16 individuals in each population, in order to focus on the more informative sites for population analyses. This approach precludes analysis of many genes with low levels of overall expression; however, we expect that many of the genes of interest for gut function should be expressed at sufficient levels for SNP analysis. Finally, we removed SNPs with a minor allele frequency <1%, as these are more likely to result from sequencing errors.

We used the software package SnpEff (version 4.2) [97] to determine whether identified SNPs were synonymous, nonsynonymous, or noncoding. Any SNPs in unigenes that were not predicted to have functional ORFs by TransDecoder were classified as noncoding. If the unigene did contain a predicted ORF, then SNPs that were located outside of the ORF region were classified as noncoding UTR variants.

### Population divergence and structure

We used the R package mmod (version 1.3.2) [98] to measure several differentiation statistics for the SNPs passing quality filters, including G_ST_ [54], G’_ST_ [99], Jost’s D [100], and ϕ_ST_ [101]. SNPs that differed significantly (α < 0.05, Bonferroni correction for multiple comparisons) between populations were identified using Fisher’s exact test using base functions in the R statistical framework (version 3.2.4) [102]. We also used a Bayesian approach implemented in BayeScan (version 2.1) [103–105] to measure F_ST_ and identify outlier SNPs.

For the set of genes with significant differentiation (G_ST_) between populations, we tested for enrichment of gene ontology terms from the GOSlim annotation. To do this, we used the Fisher exact test with false discovery rate correction implemented in Blast2GO [46–48].

We also measured average nucleotide diversity (*π*) by gene for each population separately. We calculated (*π*_*nt*_) for each nucleotide as

1 $$ {\pi}_{n t}\kern0.5em =\kern0.5em \mathbf{1}-{\displaystyle \sum_i\left(\begin{array}{c}{n}_i\\ {}2\end{array}\right)/\left(\begin{array}{c} n\\ {}2\end{array}\right)} $$

where *n*_*i*_ is the count of allele *i* in the population, and *n* = ∑*n*_*i*_. We took the average of (*π*_*nt*_) for each position across the gene as the average nucleotide diversity for each unigene.

We also calculated Tajima’s D [70] for each unigene from the nucleotide diversity and the number of SNPs within each gene. This statistic was calculated separately for each population. We took the sample size for each to be the average number of alleles genotyped at each SNP within a given locus. We defined a locus as substantially different from the neutral expectation if the value for Tajima’s D was in the upper or lower 2.5th percentile of the distribution for the population.

To examine population structure, we first performed a principal components analysis using R [102]. For this analysis, only SNPs that had genotype calls in all individuals were included. We also used fastStructure (version 1.0) [72] to explore these patterns further. All identified SNPs that passed our stringent filtering criteria were included in the fastStructure analysis. We ran the algorithm with a simple prior for values of K ranging from 1 to 10. We then used the provided script to determine the appropriate complexity for the model. The optimal value of K that maximized marginal likelihood (*K*_ε_^*^) was 2. The smallest number of components explaining the ancestry observed in the data $\left({K}_{\varnothing^C}^{\ast}\right)$ was 4. Results from both models are reported.

Finally, we used the R package related (version 1.0) [106] to estimate relatedness among individuals. Because of computational constraints, we used only the subset of SNPs for which we had genotype calls from all individuals. The triadic likelihood method [107] was used to estimate relatedness among individuals within the nonagricultural and agricultural populations separately.

## Additional files

Additional file 1: Phenotyping data for MN and ND individuals. Table containing the wing and development time measurements for all phenotyped individuals. (XLSX 18 kb)

Additional file 2: Gene ontology annotation summary. The number of annotated unigenes assigned to generic GOSlim categories from the *Pieris rapae* transcriptome are shown. (PDF 253 kb)

Additional file 3: Population statistics for all SNPs. Allele frequency and population statistics for all SNPs. (XLSX 12762 kb)

Additional file 4: Summary table of significant SNPs. Annotation and summary statistics for all SNPs significantly differentiated between MN and ND populations. (XLSX 93 kb)

Additional file 5: Summary of variable genes. Nucleotide diversity and Tajima’s D estimates by population for all genes containing SNPs. (XLSX 679 kb)

Additional file 6: Genetic relatedness within populations. Pairwise genetic relatedness in (A) the agricultural ND population and (B) the nonagricultural MN population are shown. Individuals are clustered based on the relatedness scores; fastStructure results and the date each individual egg was laid are shown to the right of each plot for comparison. (PDF 397 kb)

## Abbreviations

BLAST: Basic local alignment search tool
BUSCO: Benchmarking universal single-copy orthologues
GATK: Genome analysis toolkit
GO: Gene ontology
KEGG: Kyoto encyclopedia of genes and genomes
MN: Nonagricultural *P. rapae* population
ND: Agricultural *P. rapae* population
ORF: Open reading frame
RNA-seq: RNA sequencing
SNP: Single nucleotide polymorphism
UMGC: University of Minnesota Genomics Center
UTR: Untranslated region
