# Supplementary figures and images for: Genomic adaptation to agricultural environments: cabbage white butterflies (Pieris rapae) as a case study
Source: BMC Genomics. 2017 May 26;18:412. doi: 10.1186/s12864-017-3787-2 (PMC5446745; doi:10.1186/s12864-017-3787-2)

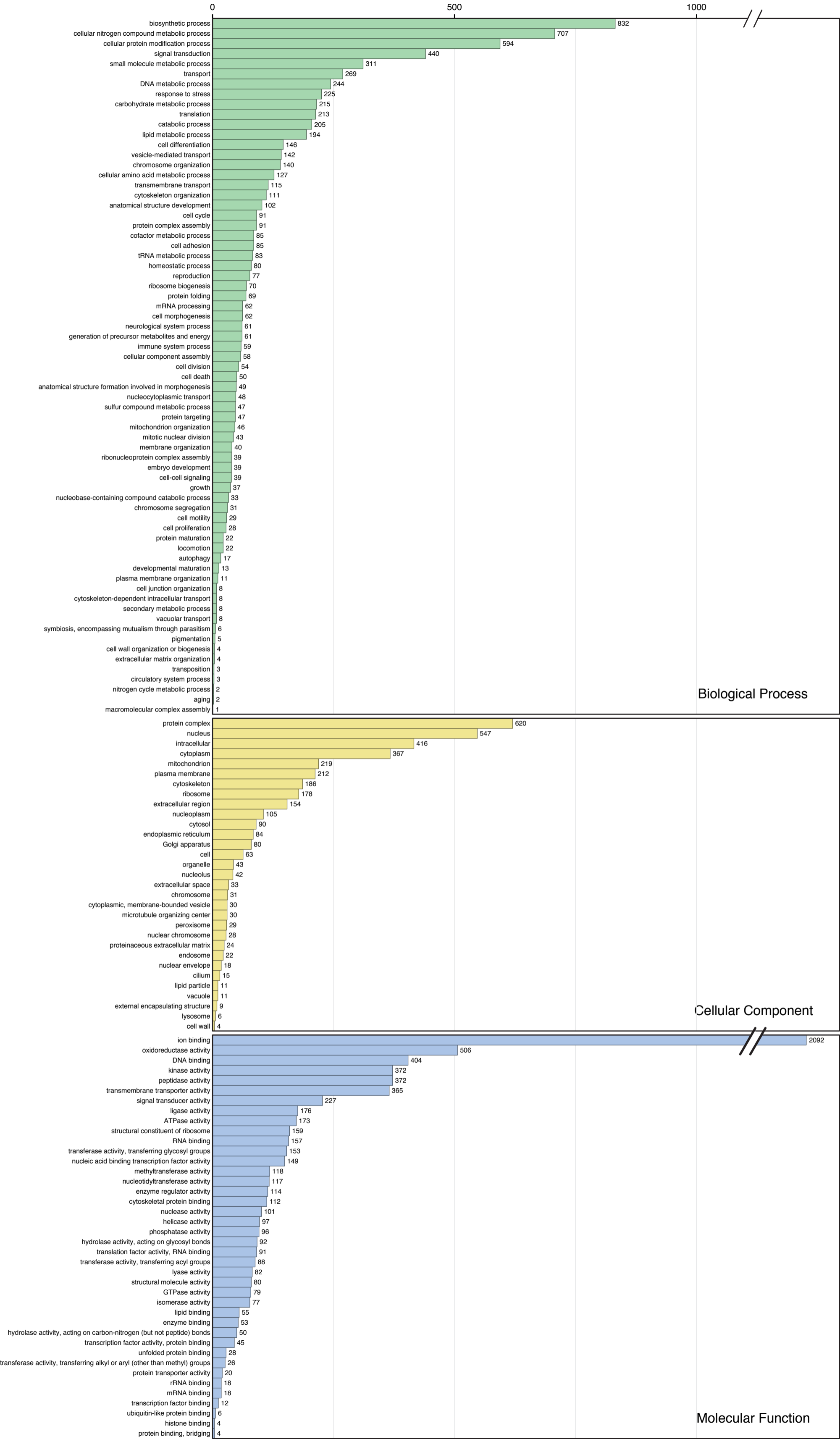

Supplement: Supplementary file 2 — Gene ontology annotation summary. The number of annotated unigenes assigned to generic GOSlim categories from the Pieris rapae transcriptome are shown. (PDF 253 kb) [file 12864_2017_3787_MOESM2_ESM.pdf]

A

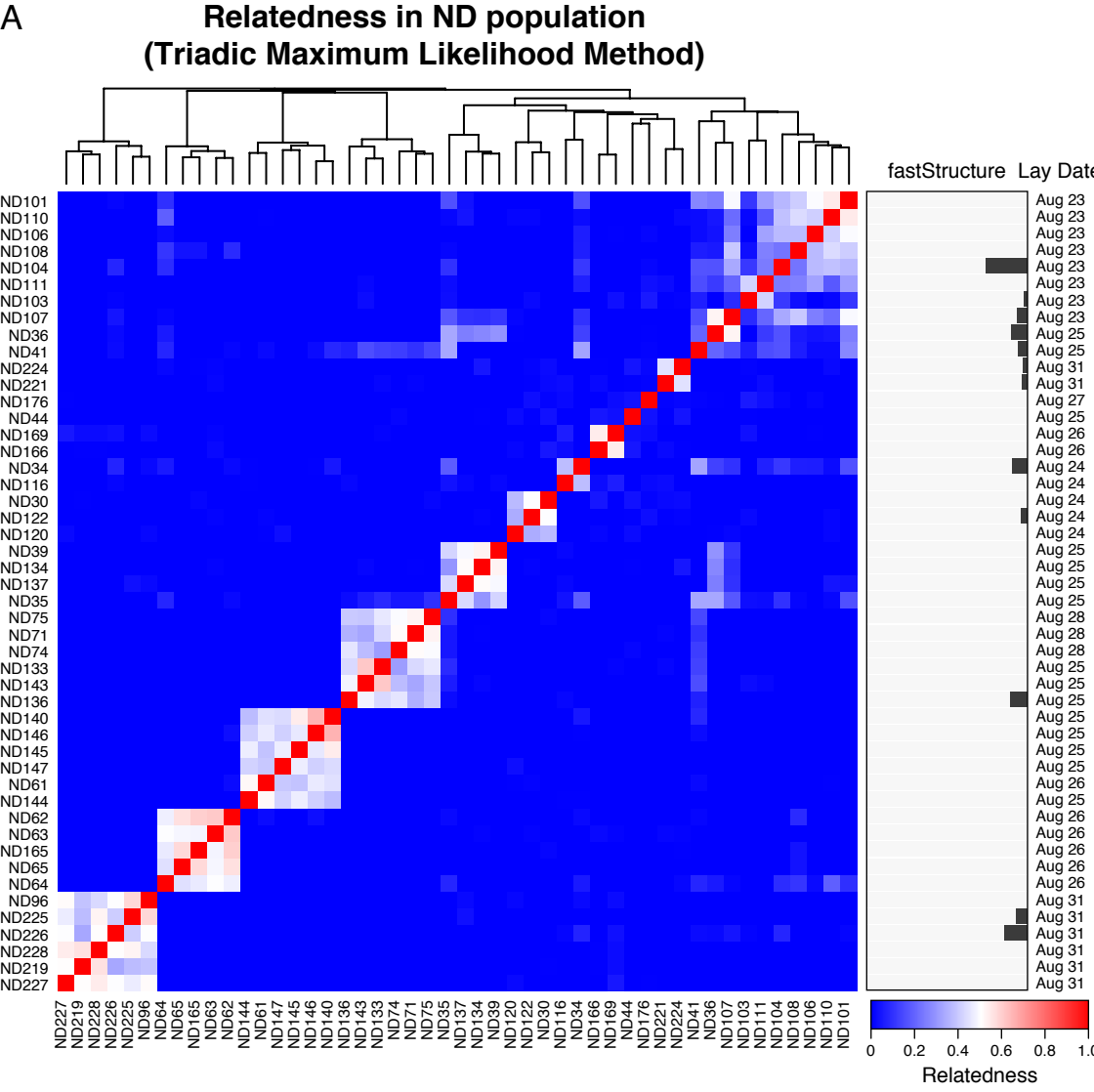

B

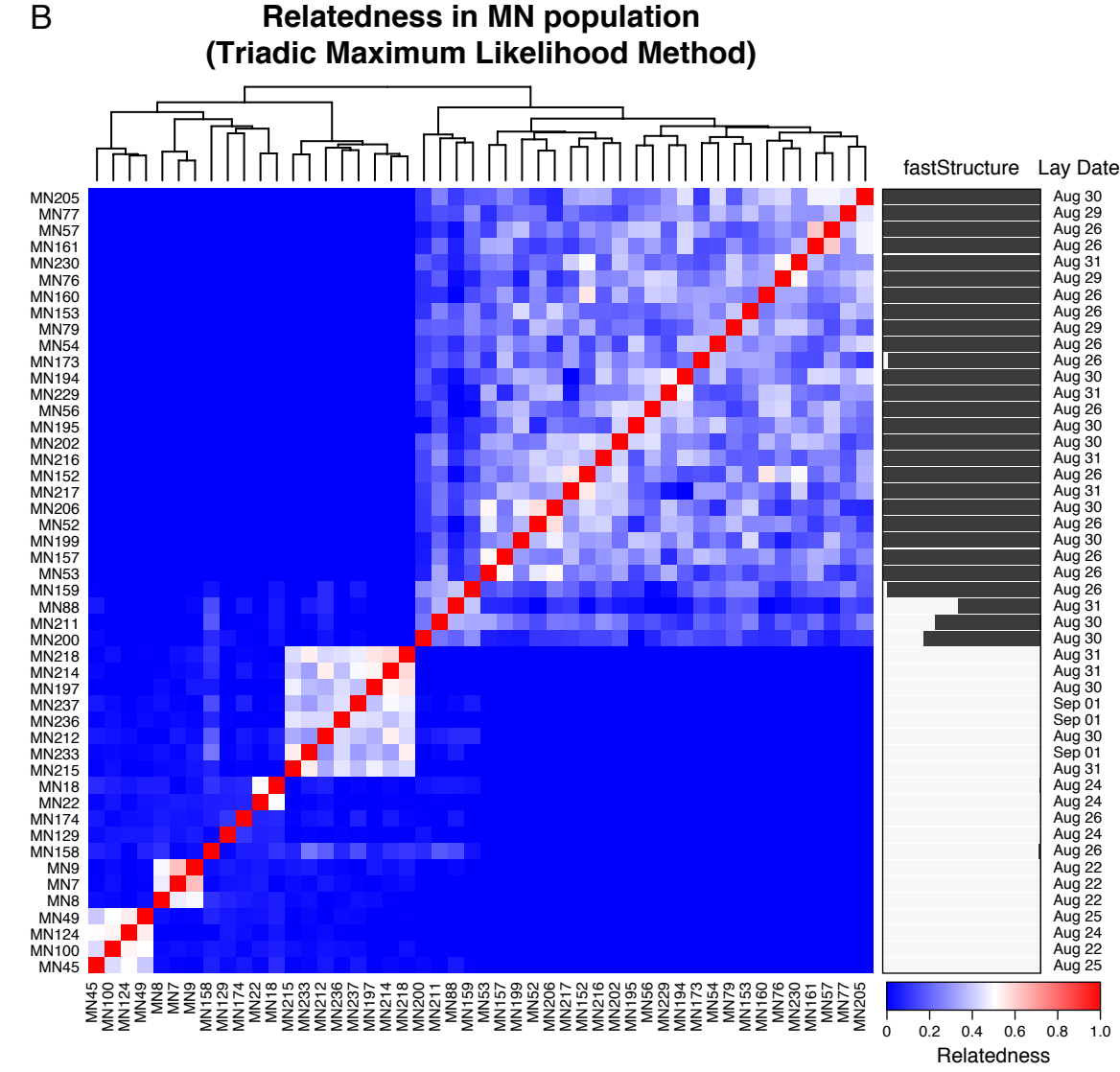

Supplement: Supplementary file 6 — Genetic relatedness within populations. Pairwise genetic relatedness in (A) the agricultural ND population and (B) the nonagricultural MN population are shown. Individuals are clustered based on the relatedness scores; fastStructure results and the date each individual egg was laid are shown to the right of each plot for comparison. (PDF 397 kb) [file 12864_2017_3787_MOESM6_ESM.pdf]
